# Wearable Devices Based on Bioimpedance Test in Heart Failure: Clinical Relevance: Systematic Review

**DOI:** 10.31083/j.rcm2509315

**Published:** 2024-09-06

**Authors:** Luis Giménez-Miranda, Santiago F. Scagliusi, Pablo Pérez-García, Alberto Olmo-Fernández, Gloria Huertas, Alberto Yúfera, Francisco J. Medrano

**Affiliations:** ^1^Internal Medicine Department, Virgen del Rocío University Hospital, 41013 Seville, Spain; ^2^Institute of Biomedicine of Seville (IBiS) - Virgen del Rocío University Hospital/University of Seville/Spanish National Research Council, 41013 Seville, Spain; ^3^Faculty of Medicine, University of Seville, 41009 Seville, Spain; ^4^Higher Technical School of Computer Engineering, University of Seville, 41012 Seville, Spain; ^5^Institute of Microelectronics of Seville - Spanish National Centre of Microelectronics (IMSE-CNM) University of Seville, 41092 Seville, Spain; ^6^Epidemiology and Public Health Networking Biomedical Research Centre (CIBERESP), 41013 Seville, Spain

**Keywords:** heart failure, bioimpedance, monitoring, wearable device, systematic review

## Abstract

**Background:**

Heart failure (HF) represents a frequent cause of hospital admission, with fluid overload directly contributing to decompensations. Bioimpedance (BI), a physical parameter linked to tissue hydration status, holds promise in monitoring congestion and improving prognosis. This systematic review aimed to assess the clinical relevance of BI-based wearable devices for HF fluid monitoring.

**Methods:**

A systematic review of the published literature was conducted in five medical databases (PubMed, Scopus, Cochrane, Web of Science, and Embase) for studies assessing wearable BI-measuring devices on HF patients following PRISMA recommendations on February 4th, 2024. The risk of bias was evaluated using the ROBINS tool.

**Results:**

The review included 10 articles with 535 participants (mean age 66.7 ± 8.9 years, males 70.4%). Three articles identified significant BI value differences between HF patients and controls or congestive vs non-congestive HF patients. Four articles focused on the devices' ability to predict HF worsening-related events, revealing an overall sensitivity of 70.0 (95% CI 68.8–71.1) and specificity of 89.1 (95% CI 88.3–89.9). One article assessed prognosis, showing that R_80kHz_ decrease was related to all-cause-mortality with a hazard ratio (HR) of 5.51 (95% CI 1.55–23.32; *p* = 0.02) and the composite all-cause-mortality and HF admission with a HR of 4.96 (95% CI 1.82–14.37; *p* = 0.01).

**Conclusions:**

BI-measuring wearable devices exhibit efficacy in detecting fluid overload and hold promise for HF monitoring. However, further studies and technological improvements are required to optimize their impact on prognosis compared to standard care before they can be routinely implemented in clinical practice.

**PROSPERO Registration:**

The search protocol was registered at PROSPERO (CRD42024509914).

## 1. Introduction

Heart failure (HF) represents a multifactorial prevalent syndrome and a frequent cause of hospitalization with significant socioeconomic impact [1, 2, 3]. The volemia status is a key factor in the pathophysiology of this disease, but unfortunately, clinical signs of congestion, such as crackles, jugular vein distention, lower limb edema, or weight gain, may not manifest until substantial volume overload occurs [4]. Timely intervention addressing congestion increases the likelihood of preventing hospital admissions [5, 6]. Given the dynamic nature of HF, monitoring and early identification of congestion are imperative for enhancing prognosis [7, 8, 9].

Multiple biotechnological approaches have been pursued, focusing on volume assessment, such as serum biomarkers (natriuretic peptides or CA-125 antigen) or biophysical parameters [10, 11, 12]. Amidst the latter, bioimpedance (BI)-frequently found in literature as bioimpedance analysis (BIA) or bioimpedance vector analysis (BIVA)-is gaining increasing attention from clinicians and researchers due to its theoretical capability to detect extracellular fluid [13].

BI measures the opposition that living tissues offer to the flow of an alternating current electrical signal. Extracellular fluid expansion, as seen in congestion, generally lead to a decrease in bioimpedance values. Previous studies involving BI assessments have mainly focused on implantable devices or punctual static measurements [14, 15]. Continuous monitoring with BI tools might offer a better and noninvasive evaluation of fluid status and variations in HF [16]. The scope of this systematic review is to delve into the clinical relevance of wearable BI-based monitoring devices for HF.

## 2. Materials and Methods

### 2.1 Search Strategy

The review process followed the Preferred Reporting Items for Systematic Reviews and Meta-Analyses (PRISMA) 2020 statement recommendations [17]. The review protocol is publicly available at PROSPERO (CRD42024509914). Research questions and search strategy were formulated using the Population, Intervention, Comparison, Outcome (PICO) framework [18]. Our population of interest was HF patients; the targeted intervention was disease monitoring through wearable impedance-measuring devices; we set the comparison as the standard care, and the outcome could be anything from BI values to weight balance, diuresis, symptoms and quality of life-related variables, readmission, or mortality. The refined search strategy was as broad as (HF OR “heart failure”) AND (impedan* OR bioimpedan* OR “phase angle” OR “BIA” OR “BIVA”) to ensure no relevant paper was missed.

### 2.2 Literature Search 

Relevant documents were searched using PubMed, Embase, Scopus, Cochrane, and Web of Science databases. The search, conducted on February 4th, 2024, was limited to original articles and randomized controlled trials published from year 2000 up to date and written in English.

### 2.3 Article Selection Process 

Results from all five literature searches were exported to .csv files and processed with Microsoft Excel, Version 2404 (Microsoft Corp., Redmond; Washington, USA). VLOOKUP function facilitated the identification and exclusion of duplicate records. Screening was independently performed by LGM and SFS, with the intervention of a third researcher (FJMO) in cases of discordance. Initial screening was performed, examining only the article title. The second screening included full-text accessibility (provided by the institutions of the researchers) and compliance with the inclusion criteria (PICO framework described above). The Risk of Bias in Non-randomized Studies (ROBINS) tool was employed to assess the quality of the selected studies [19, 20]. When two or more records explored the same cohort of subjects, the most relevant study, according to the review’s objectives, was selected. Additionally, relevant articles cited in the screened studies were sought and included if they met the inclusion criteria.

### 2.4 Data Extraction, Presentation, and Analysis

The selected studies were fully reviewed by the authors in this paper, collecting and tabulating the information regarding the first author(s) and year of publication, study design, characteristics of the population, subjects included in the analysis, wearable BI tool, outcome, main results, and risk of bias. The software utilized for the statistical parameters, such as global mean, standard deviation (SD), or 95% confidence interval (95% CI), and analysis was IBM SPSS Statistics for Windows, Version 26.0 (IBM Corp., Armonk, NY, USA).

## 3. Results

The database search yielded 5679 records, with 3975 identified as duplicates. Among the remaining 1704 records evaluated by title, 207 progressed to further screening. Only two articles could not be retrieved, leaving 205 records for detailed assessment. Only 10 articles demonstrated sufficient compliance to be included in the review.

**Fig. 1.**
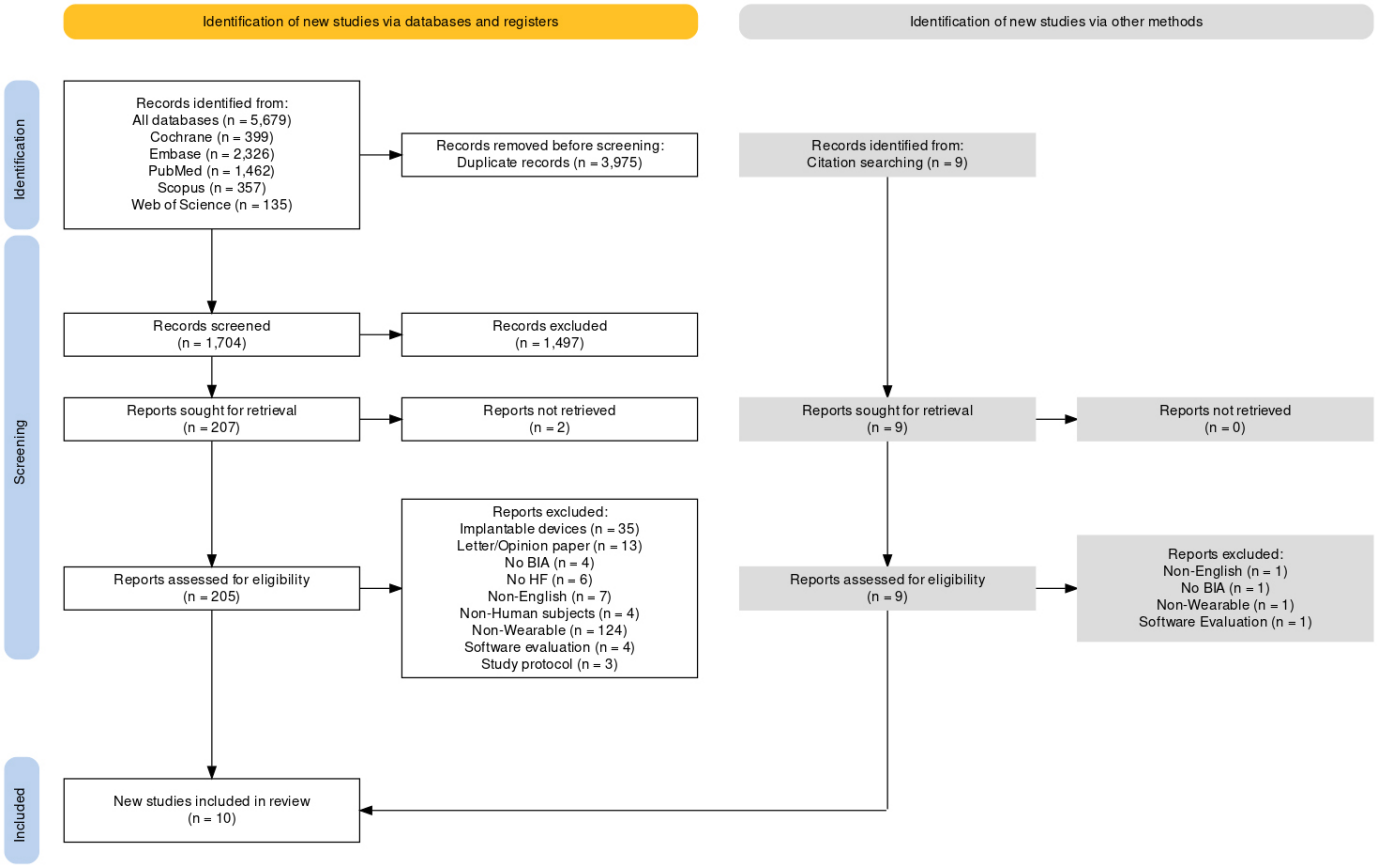
**PRISMA flow diagram**. The article selection process shows the different steps and reviewing processes. The diagram was generated using PRISMA 2020 ShinyApp [21]. BIA, bioimpedance analysis; HF, heart failure; PRISMA, Preferred Reporting Items for Systematic Reviews and Meta-Analyses.

The primary reasons for exclusion were as follows (ordered by frequency): non-wearable devices (124), invasive implantable devices (35), letter/opinion papers (13), non-English articles (7), study population not affected by heart failure (6), non-bioimpedance-based wearable devices (4), pre-clinical studies (4), software evaluation studies (4), and study protocols (3). Often, the same study had multiple published articles exploring different aspects or presenting varied results. The article that best filled the inclusion criteria was selected in these cases. While processing the search strategy, nine additional articles were identified through citations and were scrutinized. The selection process is shown in Fig. 1 (Ref. [21]) following the prism flow diagram.

The selected ten articles were conducted in North America (United States), Europe (Belgium, Germany, and Spain), and Asia (India and Singapore) from 2012 to 2023. Most studies adopted an observational design characterized by a prospective, non-controlled, and non-randomized approach. Table 1 (Ref. [22, 23, 24, 25, 26, 27, 28, 29, 30, 31]) summarizes the characteristics of the studies included in this systematic review.

**Table 1.** **Characteristics of the 10 studies included in the systematic review**.

First author	Study design	- Analyzed subjects	Wearable BI tool	Outcome	Results	Risk of bias (ROBINS)
Year		- Average age (years)				
Citation		- Ambulatory/admitted				
		- Participating centers				
		- Average LVEF/NYHA class				
		- Follow-up				
Anand	MC, N-R, N-C, prospective	- 200	Thoracic BIA multisensory Holter (MUSIC)	HFA, diuretic up-titration or death, safety (adverse effect)	Sensitivity = 0.63; specificity = 0.93; false positive rate = 0.9/patients–year; alert-to-event time = 11.5 ± 6.0 days; severe adverse event rate = 0.004/patient–year	Some concerns
2012		- 59				
[22]		- 27 centers in USA, India, and Singapore				
		- Ambulatory				
		- 27%/3.39				
		- 90 days				
Lee–Squillace–Smeets	SC, N-R, N-C, prospective	- 3	Thoracic MF-BIA multisensory Holter (IMEC)	Fluid loss, ΔBI	Correlation between fluid loss and ΔBI; R^2^ >0.8	High risk
		- N/A				
2015		- Admitted				
[23]		- N/A				
		- N/A/N/A				
		- N/A				
Gastelurrutia–Cuba-Gyllensten	SC, N-R, N-C, prospective	- 20	Thoracic BIS multisensory vest	HFD	R_0_ on admission was 11.7 ± 7.12 Ω (*p * < 0.05) lower in patients who died	Some concerns–high risk
		- 74.7				
2016		- Admitted, then ambulatory				
[24]		- GTPUH, Badalona, Spain				
		- 37%/N/A				
		- 18 months				
Cuba Gyllensten	MC, N-R, N-C, prospective	- 91	Thoracic BIA multisensory vest	HFA	Sensitivity = 0.6; specificity = 0.96; PPV = 0.11; NPV = 0.99	Some concerns
2016		- 63				
[25]		- Ambulatory				
		- 6 clinics in Germany and Spain				
		- 31%/2.44				
		- 10 months				
Darling–Dovancescu	SC, N-R, N-C, prospective	- 57	Thoracic MF-BIA multisensory vest (SENTINEL-HF)	HFA or diuretic up-titration	Sensitivity = 0.87; specificity = 0.7; accuracy = 0.72	Low risk–some concerns
		- 67.2				
2017		- Admitted, then ambulatory				
[26]		- UMMMC, MA, USA				
		- 44%/3				
		- 75 days				
Stehlik	MC, N-R, N-C, prospective	- 74	Skin BIA multisensory patch (Vital Connect®)	HFA and non-HF/non-trauma admissions	Sensitivity = 0.76–0.88; specificity = 0.85; alert-to-admission time = 6.5–8.5 days	Some concerns
2020		- 68.4				
[27]		- Admitted, then ambulatory				
		- VAMCs UT/CA/TX/FL, USA				
		- N/A/2.35				
		- 90 days				
Smeets	SC, N-R, N-C, prospective cohorts	- 36	Thoracic SF-BIA multisensory Holter (IMEC)	ACM, HFA, ACM&HFA	Decrease in R_80kHz_ related to ACM and ACM and HFA (HR 5.51 and 4.96; *p * < 0.05)	Some concerns
2020		- 81				
[28]		- Admitted, then ambulatory				
		- ZOL, Genk, Belgium				
		- 51%/N/A				
		- 12 months				
Reljin	SC, N-R, C, prospective	- 44	Thoracic MF-BIA multisensory vest (Philips)	ΔBI and heart rate values admission-discharge and controls	Accuracy = 0.82–0.92	Some concerns – High risk
2020		- 71.9				
[29]		- Admitted				
		- UMMMC, MA, USA				
		- N/A/N/A				
		- N/A				
Sanchez-Perez–Berkebile	SC, N-R, N-C, prospective	- 8	Thoracic BIS multisensory Holter	Δ*K* (R_5–150 kHz_ ratio) admission–discharge	Δ*K *= 0.05 ± 0.19; *p * < 0.001	Some concerns–high risk
2022		- 50.2				
[30]		- Admitted				
		- GMH Atlanta, GA, USA				
		- N/A/N/A				
		- N/A				
Scagliusi	SC, N-R, C, prospective	- 2	BIS Anklet (IMSE)	ΔBI	N/A	High risk
2023		- 69.5				
[31]		- Admitted				
		- VRUH, Seville, Spain				
		- N/A/N/A				
		- 30 days				

ACM, All-cause mortality; BI, bioimpedance; BIA, bioimpedance analysis; BIS, bioimpedance spectroscopy; CA, California; FL, Florida; GA, Georgia; GMH, Grady Memorial Hospital; GTPUH, German Trias Pujol University Hospital; HFA, heart failure-related admission; HFD, heart failure-related death; HF, heart failure; IMEC, Interuniversity Microelectronic Center; IMSE, Institute of Microlectronics of Seville; LVEF, left ventricular ejection fraction; MA, Massachusetts; MF-BIA, multi-frequency bioimpedance analysis; MUSIC, multisensor monitoring in congestive heart failure; N/A, not available; N-C, non-controlled; N-R, non-randomized; NPV, negative predictive value; NYHA, New York Heart Association; PPV, positive predictive value; R, resistance; ROBINS, Risk Of Bias In Non-randomized Studies; SC, single-center; SF-BIA, single-frequency bioimpedance analysis; TX, Texas; UMMMC, University of Massachusetts Memorial Medical Center; USA, United States of America; UT, Utah; VAMCs, Veteran Affairs Medical Centers; VRUH, Virgen del Rocío University Hospital; ZOL, Ziekenhuis Ost-Limburg; MC, multicentric; HR, hazard ratio.

The study populations were diverse, comprising a heterogeneous mix of exclusively admitted HF patients (some due to HF and some due to any other cause), initially admitted and later transitioning to ambulatory HF patients, exclusively ambulatory HF patients, and non-HF patients (controls). Analyzed subjects ranged from 2 to 200 per study with a median of 40, gathering 535 individuals. The average age, calculated based on available information, was 66.7 ± 8.9 years, and males constituted 70.4% of the participants. Left ventricular ejection fraction (LVEF) and New York Heart Association (NYHA) functional classification values were reported for 75.5% and 41.5% of the subjects, with mean ± SD values of 32.9 ± 8.1% and 2.5 ± 0.3, respectively. The mean follow-up duration, available for 89.7% of the analyzed subjects, was 168.3 ± 130.6 days.

The preferred BI-based wearable devices in eight of the ten studies were vests or Holter-like setups designed to assess transthoracic BI [22, 23, 24, 25, 26, 28, 29, 30]. These devices typically featured four electrodes and measured BI at multiple frequencies. In one of the studies, a two-electrode patch was employed to assess skin BI, while another study utilized a four-electrode anklet to investigate the segmental impedance of the leg, specifically targeting the identification of edema [27, 31].

The outcomes and their definitions displayed notable diversity across the included studies. Many of them, small proof-of-concept prospective studies, aimed to explore the changes in BI in HF-admitted patients undergoing depletive therapy. In 2015, Lee, Squillace, and Smeets [23] found a strong negative correlation in three HF-admitted patients between fluid balance and BI (R^2^ = 0.84 ± 0.03). In 2020, Reljin *et al*. [29] collected the transthoracic BI parameters and heart rate variability from the SHIELD study on 44 admitted patients to develop an algorithm that demonstrated an accuracy of 92% in identifying lung congestion. Two years later, Sanchez-Perez and Berkebile [30] observed changes in the resistance at 5 kHz (R_5kHz_) and 150 kHz (R_150kHz_) ratio, *K* (R_5kHz:150kHz_) assessed through thoracic BI in eight HF patients from admission to discharge, finding a significant increase in *K* of 0.05 ± 0.19 (*p* < 0.001).

Four articles focused on event prediction in relation to HF worsening with BI as an early diagnostic tool. Boasting the largest sample size within the review, in 2012, the MUSIC investigators presented an algorithm based on the BI data along with two other physiological parameters (breath index and personalized fluid status) that could predict HF-worsening related events (admission, diuretic up-titration or death) 11.5 ± 6.0 days ahead with a sensitivity of 63%, a specificity of 93%, and a false positive rate of 0.9 per patient–year [22]. Using a similar approach, in 2016, Cuba-Gyllensten *et al*. [25] published an enhanced algorithm using only transthoracic-BI that yielded a sensitivity of 60%, a specificity of 96%, and positive and negative predictive values of 10.9% and 99.6%, respectively, outperforming similar algorithms based on weight measurements in terms of predicting HF admissions. One year later, Darling and Dovancescu and colleagues [26], using the BI data from the SENTINEL-HF study and considering the events as diuretic up-titration or HF-related admission, produced with a sensitivity of 87%, specificity of 70%, and an overall accuracy of 72% within the 30-day window before the event. In 2020, the LINK-HF multicenter study that combined skin BI with physical activity and heart and respiratory rates measured using a patch showed a sensitivity of 76% to 88% and a specificity of 85% for detecting re-admissions after HF hospitalization with an alert-to-admission time of 6.5–8.5 days [27].

With this information, without forgetting the diverse nature of the studies and outcomes, we could summarize that the overall sensitivity and specificity described so far in the literature on predicting HF-related events using wearable BI—along with other parameters—measuring devices are around 70.0 (95% CI: 68.8–71.1) and 89.1 (95% CI: 88.3–89.9), respectively. Addressing mortality, apart from the aforementioned MUSIC study, in 2016, Gastelurrutia and Cuba-Gyllensten [24] followed 20 initially admitted HF patients for 18 months after discharge and observed that R_0_ (theoretical resistance value at 0 Hz frequency obtained through the Cole-Cole model [32]) on admission was 11.7 ± 7.12 Ω (*p* = 0.003) lower in patients who died during follow-up. In 2020, Smeets and colleagues [28] studied 36 patients admitted for HF in a coronary care unit, dividing them into two groups depending on whether their transthoracic BI had increased or decreased by discharge. Following discharge, they monitored them for the subsequent 12 months and performed a survival analysis that proved that a decrease in R_80kHz_ was related to all-cause-mortality with a hazard ratio (HR) of 5.51 (95% CI: 1.55–23.32; *p* = 0.02) and to the composite outcome all-cause-mortality and HF admission with a HR of 4.96 (95% CI:1.82–14.37; *p* = 0.01) [28].

## 4. Discussion

In this systematic review, we performed a comprehensive literature research study considering many document types beyond randomized control trials (as we even searched for and found conference papers). The rationale behind this approach was to avoid missing any pertinent insight and to minimize publication bias. However, we understand that certain studies, particularly those associated with industrial development and patent protection, might remain unpublished or inaccessible through our literature search scheme. It is also noticeable that most of the articles included in the review are small-size proof-of-concept studies that present serious concerns while exploring the risk of bias, and no randomized controlled trials were found; hence, conclusions drawn in this review must be cautiously and carefully considered.

Although BI has demonstrated its efficacy in detecting fluid overload and holds substantial evidence in HF management, its integration into clinical practice still faces several challenges [13]. While exploring this parameter, authors commonly find issues with inter- and intra-individual variability and confounding factors such as sex, circadian changes, body composition, position, medications, and skin abnormalities, among others. These factors contribute to the complexity of establishing a consistent reference point for BI measurements, making it challenging to interpret and apply these data in a standardized manner [14].

In a recent meta-analysis, intrathoracic BI data obtained through implantable devices failed to improve HF-related events (HF admission and all-cause mortality) predictions compared with standard care or noninvasive telemonitoring [15]. To interpret these results, the authors propose that pulmonary congestion might manifest as a late-onset feature in HF decompensation, adopting a pathophysiological approach. However, an alternative consideration could be the limited coverage area for impedance exploration provided by these devices. In contrast to implantable devices, wearable devices may offer two primary advantages: noninvasiveness and a broader coverage area for exploration.

Despite the advantages of wearable devices, such as noninvasiveness and continuous monitoring, there are still challenges to be addressed before seamless integration into clinical practice. In the reviewed papers, it is evident that over the years, together with technological development, certain technical issues, such as the quality of measures, signal processing, and the effectiveness of contact electrodes, have improved. However, there remains a need for continued implementation and refinement of this technology to potentially revolutionize the paradigm of HF monitoring and early prediction of decompensations.

Healthcare providers need to consider that the global estimated cost of HF care is USD 108 billion per year, with at least 60% of the cost directly related to admissions. Given these substantial costs, investing in tools that may reduce admissions could be a wise decision for improving prognosis and financially [33].

## 5. Conclusions

Wearable devices that measure BI have demonstrated effectiveness in detecting fluid overload and show promise in monitoring HF. However, additional studies are warranted to investigate their potential utility in predicting related events that worsen HF, thereby improving overall prognosis. Further research, including randomized controlled trials in this domain, will contribute to a more comprehensive understanding of the capabilities and clinical implications of these devices in the context of HF management.

## Availability of Data and Materials

The review protocol is publicly available at PROSPERO (CRD42024509914). The articles analyzed during the current study are available through Embase (https://www.embase.com), Cochrane (https://www.cochranelibrary.com), PubMed (https://pubmed.ncbi.nlm.nih.gov), Scopus (https://www.scopus.com) and Web of Science (https://webofscience.com) databases following the steps described in Methods but its access may be restricted depending on researcher’s institution subscriptions.

## Supplementary Material

Supplementary material associated with this article can be found, in the online version, at https://doi.org/10.31083/j.rcm2509315.
